# Histopathologic Features of *Mycobacterium ulcerans* Infection

**DOI:** 10.3201/eid0906.020485

**Published:** 2003-06

**Authors:** Jeannette Guarner, Jeanine Bartlett, Ellen A. Spotts Whitney, Pratima L. Raghunathan, Ymkje Stienstra, Kwame Asamoa, Samuel Etuaful, Erasmus Klutse, Eric Quarshie, Tjip S. van der Werf, Winette T.A. van der Graaf, C. Harold King, David A. Ashford

**Affiliations:** * Centers for Disease Control and Prevention, Atlanta, Georgia, USA; † Groningen University Hospital, the Netherlands; ‡ Ministry of Health, Accra, Ghana; § St. Martins Catholic Hospital, Agroyesum, Ghana; ¶ Dunkwa Government Hospital, Dunkwa, Ghana; # Presbyterian Hospital, Agogo, Ghana; ** Emory University, Atlanta, Georgia, USA

**Keywords:** Mycobacterium ulcerans, Buruli ulcer, histopathology, PCR, culture, acid-fast bacilli, research

## Abstract

Because of the emergence of Buruli ulcer disease, the World Health Organization launched a Global Buruli Ulcer Initiative in 1998. This indolent skin infection is caused by *Mycobacterium ulcerans*. During a study of risk factors for the disease in Ghana, adequate excisional skin-biopsy specimens were obtained from 124 clinically suspicious lesions. Buruli ulcer disease was diagnosed in 78 lesions since acid-fast bacilli (AFB) were found by histopathologic examination. Lesions with other diagnoses included filariasis (3 cases), zygomycosis (2 cases), ulcerative squamous cell carcinomas (2 cases), keratin cyst (1 case), and lymph node (1 case). Thirty-seven specimens that did not show AFB were considered suspected Buruli ulcer disease cases. Necrosis of subcutaneous tissues and dermal collagen were found more frequently in AFB-positive specimens compared with specimens from suspected case-patients (p<0.001). Defining histologic criteria for a diagnosis of Buruli ulcer disease is of clinical and public health importance since it would allow earlier treatment, leading to less deforming sequelae.

*Mycobacterium ulcerans* produces an indolent cutaneous infection known as Buruli ulcer disease (*1*–*4*). Three clinical stages of lesions have been described: preulcerative (which can present as a nodule, papule, plaque, or edema), ulcerative, and healed (scar) disease (*5*). The clinical characteristics of these lesions are nonspecific, particularly during the preulcerative stage. Lesions usually start as a single, painless, subcutaneous nodule, ill-defined edema, or plaque that enlarges over time. The skin that covers the nodule or plaque eventually sloughs off, together with the underlying tissues, forming an ulcer. If left untreated, the ulcer enlarges and becomes undermined. The patient usually has no systemic symptoms. Spontaneous healing of the ulcer has been described; healing starts at the proximal end of the ulcer and extends to the distal portions, resulting in a depressed scar that contracts and may produce severe deformities (*1*).

Many diseases can be confused with Buruli ulcer disease in each of its clinical stages; thus, laboratory tests and procedures can help establish the diagnosis (*4*). Such methods include culturing for *M. ulcerans* from lesion samples, testing swab samples of ulcers for acid-fast bacilli (AFB), histopathologic screening for characteristics of the disease, using polymerase chain reaction (PCR) to find bacterial DNA; or all of the above (*3*–*7*). Of the previously enumerated methods, only culture can be considered specific for detection of *M. ulcerans,* but it has a low sensitivity (*3*–*5*). Several authors have described the histopathologic changes of Buruli ulcer disease as the patients progress through the different clinical stages (*8*,*9*). However, letting a patient progress to a diagnostic ulcer is unacceptable. Thus, better defining diagnostic histopathologic features, particularly in the early stages, is of clinical and public health importance since this will allow early treatment and lead to less deforming sequelae.

*M. ulcerans* has been identified in many tropical and temperate parts of the world, and in the last decade reports of the disease have increased in several West African countries, including Ghana (*3*,*10*). As part of a study of surveillance, serodiagnosis, and identification of risk factors for Buruli ulcer disease in Ghana, excisional skin-biopsy specimens were obtained from clinically suspect lesions. We review the histopathologic examination of the skin specimens from the patients from Ghana and list the differential diagnoses encountered in preulcerative and ulcerative lesions. We compare the histopathologic features of AFB-positive and AFB-negative specimens and correlate pathologic examination, PCR, and culture results.

## Materials and Methods

A total of 144 excisional skin-biopsy specimens were obtained from patients with clinically suspect lesions at therapeutic centers of excellence for Buruli ulcer disease in three highly disease-endemic districts in Ghana. Specimens were fixed in formalin and transported to the Infectious Disease Pathology Activity at the Centers for Disease Control and Prevention, Atlanta, GA. Representative portions of the specimens, measuring about 2.5 x 1.5 x 0.5 cm, were embedded in paraffin in one cassette, and sections were stained with hematoxylin and eosin (H&E) and Ziehl-Neelsen (to highlight AFB). Table 1 describes the histopathologic features evaluated. One pathologist (JG) searched for AFB, using the 40X magnification objective through the entire section (each slide was reviewed for 45 to 60 min). When a section did not have AFB bacilli, additional tissue specimens with approximately the same dimensions as the first were embedded in paraffin and studied with H&E and Ziehl-Neelsen stains. In AFB-negative cases, fungal causes of nodules and ulcers were searched by using Grocott methenamine silver.

**Table 1:** Histopathologic features evaluated in definitive and suspected Buruli ulcer cases

Location, feature	Comments	
**Epidermis**		
Hyperplasia	Psoriasiform (regular downward elongation of rete ridges), or pseudoepitheliomatous (irregular elongation of rete ridges)	
AFB^a^	Presence or absence	
**Dermis**		
Elastolysis	Collagen degeneration and necrosis seen as granular blue/purple collagen bundles with H&E stain	
Inflammation, type	Acute (presence of neutrophils), chronic (presence of lymphocytes and macrophages), or granulomatous (presence of multinucleated giant cells and epithelioid histiocytes)	
AFB	Presence or absence	
Vascular changes	Thickening of the media, necrosis, and inflammation of vascular walls	
**Subcutis**		
Necrosis	Coagulative or fat necrosis	
Inflammation, type	Acute (presence of neutrophils), chronic (presence of lymphocytes and macrophages), or granulomatous (presence of multinucleated giant cells and epithelioid histiocytes)	
Inflammation, intensity	Absent, mild (scattered inflammatory cells), or intense (inflammation forming nodules or bands)	
AFB	Absent, mild (1–5 AFB seen with 40X objective), moderate (>6 AFB seen with 40X objective), or marked (AFB seen with 20X objective as clumps or colonies)	

^a^H&E, hematoxylin and eosin stain; AFB, acid-fast bacilli.

Cases in which the specimen lacked subcutaneous adipose tissue were excluded from analysis. Specimens in which AFB were found in histologic sections were considered definite cases; specimens with negative AFB were considered suspected cases unless they had other diagnoses that could account for a clinical nodule or ulcer. Confirmation of histopathologic diagnosis of Buruli ulcer disease was possible in 62 cases by using culture, PCR for *M. ulcerans,* or both. Culture and PCR (IS2404) were performed according to standard techniques (*4*,*11*). Complete details of these techniques and a comparison of diagnostic methods will be published separately.

The frequency of diagnosis of nodules versus diagnosis of ulcers was determined. The histopathologic features of AFB-positive specimens compared to such features in AFB-negative specimens, and definite Buruli ulcer disease preulcerative lesions compared to ulcerative lesions were derived with SAS 8.2 (SAS Institute Inc., Cary, NC) by using the chi-square and Fisher exact tests with a statistical significance of p=0.05. The same statistical tools were used to analyze frequencies of the histopathologic features in AFB-negative versus confirmed cases.

## Results

Adequate material for histopathologic review was available for 124 of 144 cases. Twenty cases (14%) were excluded because of lack of subcutaneous tissue in the biopsy specimen. The results of analysis of these 124 specimens are shown in Table 2. In summary, using histopathologic methods, we evaluated 30 (24%) nodules, 6 (5%) plaques, and 88 (71%) ulcers. By histopathologic examination, diagnoses other than Buruli ulcer disease were found for nine patients, seven of whom had nodules and two of whom had ulcers. Patients with nodules included three with parasites (two *Onchocerca volvulus* and one *Mansonella streptocerca*), two with deep fungi (subcutaneous zygomycosis), one with a keratin cyst, and one with a hyperplastic lymph node; the two patients with ulcers had squamous cell carcinoma. AFB were present in 78 (63%) specimens, while 37 (30%) specimens did not have AFB and were considered suspected cases of Buruli ulcer disease. Of 78 cases with positive AFB, bacilli were found in 69 (88%) of the first specimens submitted for pathology and 9 (11%) when additional tissue (available in 48 of the original AFB-negative specimens) was studied. We had a histopathologic diagnosis in 25 (83%) of the 30 nodules, whether this was definite Buruli ulcer disease or another diagnosis; the proportion of pathology diagnosis was lower for ulcers (57 [65%] of 88). The proportion of nodules and ulcers with a definite diagnosis of Buruli ulcer disease was approximately the same (60% vs. 62%).

**Table 2:** Number (percent) of specimens with other diagnoses, definite, and suspected Buruli ulcer according to clinical stage^a^

Clinical stage	Other diagnoses	Definite BU (AFB positive)	Suspect BU (AFB negative)	Total
Nodule	7 (6)	18 (14)	5 (4)	30 (24)
Plaque	0	5 (4)	1 (0.8)	6 (5)
Ulcer	2 (1.6)	55 (44)	31 (25)	88 (71)
Total	9 (7)	78 (63)	37 (30)	124 (100)

^a^BU, Buruli ulcer; AFB, acid-fast bacilli.

The histopathologic features for AFB-positive and AFB-negative cases are shown in Table 3. Necrosis of the subcutaneous tissues was found in 100% of AFB-positive and 62% of AFB-negative cases (p<0.001). Sixty-one percent of AFB-positive cases and 6% of AFB-negative cases had necrotic collagen in the dermis (elastolysis) (p<0.001). In 92% of AFB-positive cases, the inflammatory infiltrate had neutrophils mixed with mononuclear cells; by contrast, suspected cases had a predominance of mononuclear inflammation (p=0.008). Epidermal hyperplasia (either psoriasiform or pseudoepitheliomatous), chronic and granulomatous inflammation, and vasculopathy were found at approximately the same rate for both AFB-positive and -negative cases. Duration of the lesion was available for 113 cases; 70 were positive for AFB, 35 were negative, and 8 had other diagnoses. Forty-seven (67%) AFB-positive cases had lesions that had been present for <3 months, with a median of 2 months (range 0.2–36); for AFB-negative cases, 19 (54%) had lesions that were present <3months, with a median of 3 months (range 0.2–156) (p=0.64).

**Table 3:** Comparison of histopathologic features of definite and suspected Buruli ulcer cases

Histopathologic feature	Buruli ulcer (AFB positive)^a^ No. (%)	Suspected Buruli ulcer (AFB negative)^a^ no. (%)	p value
**Epidermis** ^b^			
Hyperplasia	50/73 (68)	21/35 (60)	0.38
**Dermis** ^b^			
Elastolysis	45/74 (61)	2/35 (6)	<0.0001
**Subcutaneous tissue**			
Necrosis	78/78 (100)	23/37 (62)	<0.0001
Vasculopathy	58/78 (74)	27/37 (73)	0.87
Acute inflammation^c^	72/78 (92)	27/37 (73)	0.008
Chronic inflammation^d^	31/78 (40)	18/37 (49)	0.36
Granulomas^e^	30/78 (38)	12/37 (32)	0.53

^a^AFB, acid-fast bacilli. ^b^Seven specimens did not have epidermis, and six did not have dermis. ^c^Acute inflammation considered as present versus absent. ^d^Chronic inflammation considered as intense versus mild. ^e^Granulomas considered as present versus absent.

**Figure 1:**
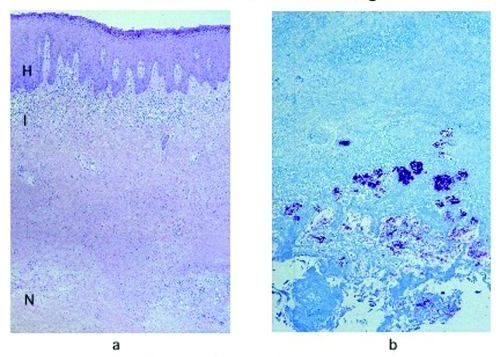
a, Hematoxylin and eosin stain of a lesion specimen showing definitive Buruli ulcer disease in the preulcerative stage (original magnification 50x). Notice the psoriasiform epidermal hyperplasia (H), superficial dermal lichenoid inflammatory infiltrate (I), and necrosis of subcutaneous tissues (N). b, Ziehl-Neelsen stain of the same nodule, showing abundant colonies of acid-fast bacilli in the necrotic subcutaneous tissues (original magnification 100x).

**Figure 2:**
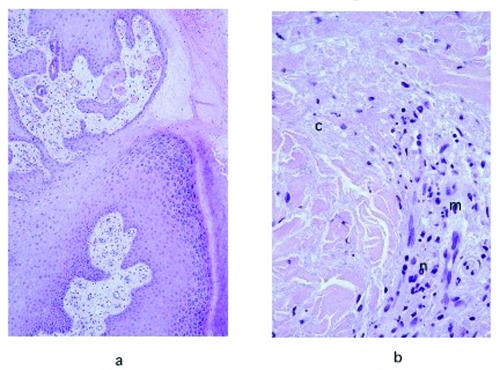
a, hematoxylin and eosin stain of the pseudoepitheliomatous hyperplasia of the epidermis in a lesion specimen showing definitive Buruli ulcer disease in the ulcerative stage (original magnification 100x). b, hematoxylin and eosin stain of the necrotic collagen (c) accompanied by mild inflammatory infiltrate in the dermis of a definitive Buruli ulcer disease lesion in the ulcerative stage (original magnification 400x). n, neutrophis; m, mononuclear cells.

The frequency of the histopathologic variables for preulcerative and ulcerative stage of AFB-positive cases is shown in Table 4. Variables that showed significant association with the ulcerative stage included epidermal hyperplasia (p=0.005), intense chronic inflammation (p=0.013), and granulomas (p=0.005). Dermal elastolysis was more frequent in preulcerative lesions (p=0.015). Of note is the lack of a statistically significant difference between preulcerative and ulcerative lesions for the concentration of AFB in the subcutaneous tissues (p=0.07). Psoriasiform epidermal hyperplasia was found in 47 Buruli ulcer disease cases, 7 in preulcerative lesions and 40 in ulcers. Pseudoepitheliomatous hyperplasia was found in 27 cases, 3 with preulcerative lesions and 24 with ulcers. AFB in the keratin were found in one nodule and in seven ulcer cases. Figure 1 shows a photomicrograph of a nodule, and Figure 2 shows an ulcer from a definitive case.

**Table 4:** Comparison of histopathologic features of preulcerative and ulcerative lesions in definite Buruli ulcer cases

Histopathologic feature	Preulcerative no. (%)	Ulcerative no. (%)	p value	
**Epidermis**	8 (42)	42 (78)	0.005	
Hyperplasia^a^				
**Dermis**	17 (85)	28(52)	0.015	
Elastolysis^a^				
AFB in dermis^b^	8 (40)	10 (19)	0.34	
**Subcutaneous tissue**				
AFB in subcutis^c^	18 (78)	31 (56)	0.074	
Acute inflammation^d^	22 (96)	50 (91)	0.48	
Chronic inflammation^e^	4 (17)	27 (49)	0.013	
Granulomas^f^	3 (13)	27 (49)	0.005	

^a^Two specimens did not have epidermis, and one did not have dermis. ^b^Considered as presence of acid-fast bacili (AFB) in dermis. ^c^Considered as intense versus mild AFB in subcutis. ^d^Acute inflammation considered as present versus absent. ^e^Chronic inflammation considered as intense versus mild. ^f^Granulomas considered as present versus absent.

Of the 37 AFB-negative cases, 1 had a positive culture, and 22 had *M. ulcerans* nucleic acids detected by PCR. However, positive PCR results were also obtained from samples of cases with filarial nodules (three patients), keratin cyst, deep fungi, and squamous cell carcinoma (one patient each). Analysis of hsitopathologic features showed a significant association of necrosis of subcutaneous tissues and elastolysis (p=0.0009 and p<0.0001, respectively) with confirmed cases compared to AFB-, culture-, and PCR-negative cases.

## Discussion

Necrosis of subcutaneous tissues and dermal collagen accompanied by minimal inflammation and AFB are considered the most reliable histopathologic features for the diagnosis of Buruli ulcer disease (*8*,*9*,*12*). Our study demonstrated that necrosis of both the subcutis and dermal collagen was significantly associated with cases. The necrosis found in such cases has been attributed to a polyketide (called mycolactone) that is produced by *M. ulcerans* and acts as an extracellular toxin (*13*–*16*). AFB-positive cases showed a significant association with the presence of neutrophils mixed in the necrotic material. In Buruli ulcer disease cases, inflammation appears to be minor for the amount of necrosis, which accounts for previous descriptions of minimal inflammatory response. Possibly, the toxin that induces adipose cell necrosis also induces necrosis of the inflammatory infiltrate.

Several authors have established that during the preulcerative stage and early in the ulcerative stage, the coagulative necrosis forms a nidus where calcifications and AFB colonies are easily visualized (*8*,*9*,*12*,*17*). However, when the ulcer starts healing, and granulation tissue, fibrosis, and granulomatous inflammation are present, AFB are difficult to document (*8*,*9*,*17*). Our study showed AFB in the keratin layer in seven of the Buruli ulcer disease ulcer cases and only in one nodule. AFB in keratin have been observed previously and may represent bacilli from colonies that are actively being sloughed off or they may be carry over from histologic processing (*8*). Contrary to findings in published reports, our study did not show a statistically significant difference in the amount of AFB in the subcutaneous tissues in the preulcerative and ulcerative stages. Additionally, the presence of AFB in clinically suspect lesions was not related to the duration of the lesion. These findings can be explained by any or all of the following factors: our study had a small number of cases in the preulcerative stage; our sampling techniques were geared to maximizing the amounts of AFB in the tissues (AFB have been observed more frequently in the distal portion of the ulcer); we obtained lesion samples at an earlier stage than other researchers. In addition, previous studies have not used statistical methods to analyze the frequency of histopathologic features; thus, the interpretation of results has been subjective.

Our study showed that epidermal hyperplasia and chronic inflammation with formation of granulomas were statistically more frequent in Buruli ulcers than in preulcerative lesions. All these features have been described to be more prominent in the later stages of disease (*8*,*9*). Epidermal regeneration occurs in an effort to cover the epidermal tissue defect (*18*). In our cases, the most frequent type of regeneration was psoriasiform, with some case-patients exhibiting pseudoepitheliomatous hyperplasia. Granulomatous inflammation is characteristic of persistent infections that evoke delayed hypersensitivity, as seen in several mycobacterial and fungal infections. Some authors have suggested that leprosy and Buruli ulcer disease may have similar gradation of the inflammatory process from foamy macrophages (lepromatous) to well-formed granulomas (tuberculoid) (*9*,*19*).

Currently, confirmation of clinically suspected cases of Buruli ulcer disease is based on culture of *M. ulcerans* from the tissues, presence of AFB in swab samples, evidence of *M. ulcerans* DNA, or characteristic histopathologic changes in tissue sections (*3*,*4*). Several problems are evident from this manner of confirming a diagnosis. The isolation rate of *M. ulcerans* from patients approaches only 35% because the bacteria are very difficult to culture (*20*). In our series, only one suspected case was confirmed by using culture. AFB in swab samples are rare and depend on the clinical stage and tissue sampled (*3*,*4*,*8*,*20*). None of the cases in this cohort was confirmed by AFB in swab specimens. PCR would conceivably be helpful, but no data are available on sensitivity and specificity with large numbers of clinical specimens (*5*,*11*). In this cohort, PCR showed positive results in cases with filarial nodules and a keratin cyst; since these patients had single nodules and did not have histopathologic evidence of Buruli ulcer disease, the findings probably represent false-positive PCR results. The cases with positive PCR results and squamous cell carcinoma and deep fungi could potentially have either two concomitant infections or a cancer arising from long-standing Buruli ulcer disease (*21*). Issues of DNA contamination, required technical expertise, and PCR costs prohibit this assay’s utility as a routine clinical diagnostic tool in the field (*3*,*5*,*13*,*14*).

“Characteristic” histopathologic changes are considered one of the confirmatory laboratory methods for Buruli ulcer disease; however, the features are nonspecific and change as the lesion evolves from a nodule to an ulcer. In this study, definite histopathologic diagnosis of Buruli ulcer disease was only possible in 63% of cases because the presence of AFB bacilli was not always detectable even though necrosis of subcutaneous tissue and collagen were observed. An additional challenge in the histopathologic diagnosis of this disease is having adequate tissue samples. In our study, 14% of the specimens were considered inadequate because they lacked subcutaneous tissue, and among those with adequate material, 11% required additional tissue to demonstrate AFB. New techniques that can be applied to tissue are greatly needed to diagnose Buruli ulcer disease in all stages. Until these techniques are available, defining diagnostic histopathologic features of the disease will enable better understanding of clinicopathologic and pathogenetic characteristics of *M. ulcerans* infection.

For this study, we collected samples from more ulcers than nodules; however, a higher proportion of nodule specimens received a histopathologic diagnosis. The histopathologic differential diagnosis for ulcers included squamous cell carcinomas only; among the other clinical diagnoses that can be encountered in this stage are tropical phagedemic ulcer, actinomycosis, noma, leishmaniasis, yaws, and scrofuloderma (*4*). In our cohort, the histopathologic differential diagnosis for nodules was more extensive and included other infectious diseases (filaria and zygomycosis) as well as other noninfectious causes of skin nodules (keratin cyst) (*4*).

In summary, our study shows the histopathologic features of patients with clinically suspected Buruli ulcer disease in a population with a high prevalence of the disease. We found that necrosis of subcutaneous tissues and dermal collagen was the best predictor of disease; however, the histopathologic changes are not unique, and diagnosis requires correlation with the clinical picture and other laboratory techniques.
